# Resilience—A Concept Toward an Actionable Clinical Approach to Aging

**DOI:** 10.1111/acel.70839

**Published:** 2026-11-14

**Authors:** Vanessa Matos Gonçalves, Ekaterina Maevskaia, Moon‐Ju Kim, Adrian Molière, Sijie Ji, Ferdinand von Meyenn, Grzegorz Filip Sarek, Elisabeth Roider, Catherine R. Jutzeler, Edouard Battegay, René M. Rossi, Collin Y. Ewald, Simon Annaheim, Jörg Goldhahn, Wei Gao, Noé Brasier

**Affiliations:** ^1^ Institute for Molecular and Translational Medicine ETH Zurich Zurich Switzerland; ^2^ Department of Health Science and Technology ETH Zurich Zurich Switzerland; ^3^ Empa, Swiss Federal Laboratories for Materials Science and Technology Laboratory for Biomimetic Membranes and Textiles St. Gallen Switzerland; ^4^ Andrew and Peggy Cherng Department of Medical Engineering, Division of Engineering and Applied Science California Institute of Technology Pasadena California USA; ^5^ Nutrition and Metabolic Epigenetics, Department of Health Science and Technology ETH Zurich Zurich Switzerland; ^6^ Department of Dermatology University Hospital of Basel Basel Switzerland; ^7^ SIB Swiss Institute of Bioinformatics Lausanne Switzerland; ^8^ International Center for Multimorbidity and Complexity in Medicine, Healthy Longevity Center University of Zurich Basel Switzerland

**Keywords:** aging, clinical concept, resilience, wearable sensors

## Abstract

Aging research has increasingly recognized the need for accurate biomarkers of aging, with aging clocks emerging as a powerful tool to quantify the degree of age‐related biological change. However, broad clinical implementation and the establishment of wide use of these biomarkers of aging remain ongoing. Resilience reflects a dynamic property of physiological homeostasis and offers a complementary lens to functionally interpret aging. Resilience does not capture the ground state the biological system is in, but rather its ability to maintain or improve function when challenged by adversity. Advances in wearable sensing technologies now enable scalable, continuous, and laboratory‐independent assessment of physiological, biochemical, and environmental parameters, which potentially offer a first window into resilience. Clinically, resilience holds promise for person‐centered risk stratification and guiding interventions, particularly in multimorbid populations. As such, we propose two lines of clinical resilience: first, reflecting involvement in disease onset mechanisms, and a second representing the capability to withstand and recover from medical interventions such as surgery or systemic pharmacotherapy. Integrating resilience into clinical frameworks may support personalized, proactive, and preventive approaches to aging, ultimately leading to meaningful clinical outcomes.

## Graphical abstract

Resilience—the capacity to maintain or improve functional ability in the face of adversity—becomes clinically actionable when tied to intrinsic/extrinsic stressors. Continuous and laboratory‐independent wearable sensing could track this response as it unfolds, turning clinical resilience into a measurable trajectory in aging.

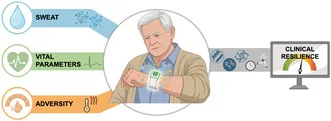

Aging in humans is characterized by the progressive decline in function on all levels of organization, from molecules to cells, tissues, and organs (Gladyshev 2016; López‐Otín et al. 2013, 2023; Kennedy et al. 2014). Although historically there were attempts to narrow down the biological aging process to individual mechanisms, for example, oxidative damage (Harman 1956), aging is now increasingly recognized as being multifactorial and highly complex (Cohen et al. 2022). This shift in thinking was formalized about a decade ago with the recognition of multiple “pillars” or “hallmarks” of aging (López‐Otín et al. 2013, 2023; Kennedy et al. 2014). Since then, ongoing biological discoveries have continually expanded the list of contributors to biological aging, with new hallmarks regularly being proposed (López‐Otín et al. 2023; Davies et al. 2022; Schmauck‐Medina et al. 2022; Knupp and Miura 2018; Manni et al. 2023; Isima and Gil 2023; López‐Otín and Kroemer 2024; Harries 2023), suggesting that complex systems approaches are necessary to fully understand the process (Cohen et al. 2022; Wensink and Cohen 2021).

A major insight from research across disciplines is that the rate of functional decline with age is not fixed, but can be modulated by targeting longevity‐associated molecular pathways, such as mTOR and insulin/IGF‐1 signaling (Greer and Brunet 2008). This plasticity of the aging process led to increased focus on the concept of biological age. Biological age is defined as the predicted age of an organism based on the level of age‐dependent biological changes, in contrast to the chronological passage of time (Moqri et al. 2023) (Table 1). Although the concept of biological age itself is not new, with researchers trying to quantify different aging rates as early as the 1960s (Hollingsworth et al. 1965), the rapid accumulation of omics data has significantly accelerated the development of new tools to measure biological age, so‐called aging clocks, based on, for example, DNA methylation, gene expression, blood biochemistry, and proteomic data (Horvath 2013; Lu et al. 2019; Levine et al. 2018; Belsky et al. 2022; Jung et al. 2023; Tyshkovskiy et al. 2024; Klemera and Doubal 2006; Cohen et al. 2013; Hwangbo et al. 2021; Krištić et al. 2014; Meyer et al. 2025; Argentieri et al. 2024; Oh et al. 2023; Petkovich et al. 2017). Although the first models were simply trained to predict chronological age, next‐generation models incorporated information on health outcomes and the pace of age‐related change (Teschendorff and Horvath 2025). Later models, such as DunedinPACE and GrimAge, have proven to capture information relevant for future health outcomes and are starting to be applied in clinical settings (Lu et al. 2019; Belsky et al. 2022; Bischoff‐Ferrari et al. 2025; Waziry et al. 2023).

**TABLE 1:** Conceptual distinctions between resilience and related constructs in aging.

Construct	Core definition	Property described	Typical operationalization	Anchor framework
Resilience	“The ability to maintain or improve a level of functional ability in the face of adversity through resistance, recovery or adaptation”	Dynamic response	Recovery trajectory after a challenge (surgery, hospitalization, infection)	WHO (2015); Hadley et al. (2017)
Frailty	“Extreme vulnerability to endogenous and exogenous stressors that exposes an individual to a higher risk of negative health‐related outcomes”	State	Fried phenotype; Rockwood deficit‐accumulation index; Clinical Frailty Scale (CFS)	WHO (2015); Fried et al. (2001); Rockwood and Mitnitski (2007)
Intrinsic Capacity (IC)	“The composite of all the physical and mental capacities that an individual can draw on”	Capacity	Key Domains: Locomotor capacity; Cognition; Vitality; Vision; Hearing; Psychological capacity	WHO (2015) (IC definition) WHO ICOPE 2nd ed. 2025 WHO (2025) (Key domains)
Functional Ability (FA)	“The health‐related attributes that enable people to be and to do what they have reason to value; it is made up of the intrinsic capacity of the individual, relevant environmental characteristics and the interactions between the individual and these characteristics”	State (composite: capacity × environment)	IC combined with environmental factors	WHO (2015)
Physiologic Reserve	“The potential capacity of a cell, tissue, or organ system to function beyond its basal level in response to alterations in physiologic demands”	Capacity	Stress‐test responses (e.g., VO_2_max, expiratory reserve volume)	Whitson et al. (2016)
Biological age	Predicted age of an organism based on the level of age‐dependent biological changes, in contrast to the chronological passage of time	State/trajectory	Epigenetic clocks; phenotypic and composite biomarker age indices	Moqri et al. (2023); Horvath (2013); Levine et al. (2018)

*Note:* The table compares resilience with frailty, intrinsic capacity, functional ability, physiological reserve, and biological age across their core definitions, the type of property each describes, their typical operationalization, and the anchor framework from which each derives. Clinical resilience, as developed here, denotes resilience assessed in relation to a defined clinical stressor; it is a specification of the resilience construct for the clinical setting rather than a distinct construct.

Despite these advances, a fundamental limitation remains: Most aging clocks are trained on cross‐sectional population‐level data and are optimized to capture features that, on average, change with age or predict adverse outcomes. As a result, they primarily estimate an individual's position along an aging continuum, or in the case of pace of aging models, the change on such a continuum (Belsky et al. 2022), reflecting the system at an average, baseline state during aging. To a degree, the ability to maintain or improve a level of functional ability in the face of adversity—resilience (WHO 2015)—may be captured by conventional aging biomarkers indirectly, as a worse baseline state in itself reduces resilience. However, a system's resilience is likely not to become clinically symptomatic in the absence of adversity such as infections or trauma; thus, differences in resilience between systems with an apparently similar baseline state might be undetectable when unperturbed (Cohen et al. 2022). Methods to assess clinical resilience during aging through dynamic responses to acute *stimuli* thus have the potential to complement established biomarkers of aging as well as provide important insights into risk/benefit assessments along clinical management, for example, in outcome prediction.

This review provides a perspective on developing the concept of clinical resilience and a translational roadmap toward actionable clinical approaches to aging. The review is structured accordingly: we define resilience and differentiate it from related constructs, develop the concept of clinical resilience, consider how wearable technologies could support its operationalization, and, finally, discuss the next steps, challenges, and opportunities this entails. We lay the foundation for embedding the concept into clinical pathways by outlining its potential added clinical value and by considering emerging wearable technologies, which can offer continuous, mostly noninvasive, and laboratory‐independent health measurements outside of controlled settings. While decoding and technology development to assess the complexity of aging is challenging, we propose that mass spectrometry‐backed wearable platforms could, in principle, provide access to develop and establish biomarkers associated with resilience during aging. If validated, a structured and evidence‐based assessment of resilience could complement functional capacity evaluation when facing adversity in clinical care and support person‐centered care across the lifespan. As an opinion‐driven narrative review, the cited literature was identified primarily through scientific sources such as PubMed and selected purposively by the authors to illustrate key concepts, rather than through a systematic search strategy.

## 1 Resilience—A Key Property of Complex Systems

Across disciplines, complex systems exhibit critical thresholds at which they can transition abruptly between states (Scheffer et al. 2009; Cohen et al. 2025), though such transitions may lead to an alternative stable state rather than collapse (Gunderson 2000). Within this context, robustness refers to the preservation of a function, whereas resilience—defined by the WHO as (“the ability to maintain or improve a level of functional ability in the face of adversity through resistance, recovery, or adaptation”)—captures a broader, dynamic process. To clarify different perspectives on resilience, three complementary interpretations have been proposed: (1) the ability to resist functional decline in response to adversity (Whitson et al. 2016, 2018); (2) the capacity to return or bounce back to homeostasis (Kirkland et al. 2016) from a physical challenge (Resnick et al. 2011), stress, or a physically stressful event (Lei et al. 2022); or (3) the ability to cope with a challenge or a stressor and its adverse effects (Colon‐Emeric et al. 2023). These map onto the three mechanisms named in the WHO definition—resistance (1), recovery (2), and adaptation (3)—and should be understood as emphasizing distinct components of a unified process rather than representing competing definitions. Accordingly, we use “resilience” to denote this integrated process.

The present work adopts the WHO framework outlined in the World Report on aging and health (WHO 2015), which situates resilience within a broader conceptual model of capacity and function in older age. To distinguish related constructs, their definitions are summarized in Table 1. In particular, intrinsic capacity (IC) is defined as “the composite of all the physical and mental capacities an individual can draw on”, whereas functional ability (FA) refers to the health‐related attributes that enable people to be and to do what they have reason to value. FA emerges from the interaction between IC and extrinsic factors, notably the individual's environment (Figure 1). Both IC and FA describe an individual's current or potential state and can be assessed in the absence of stressors, thereby characterizing the available resources and constituting the potential domain.

**FIGURE 1:**
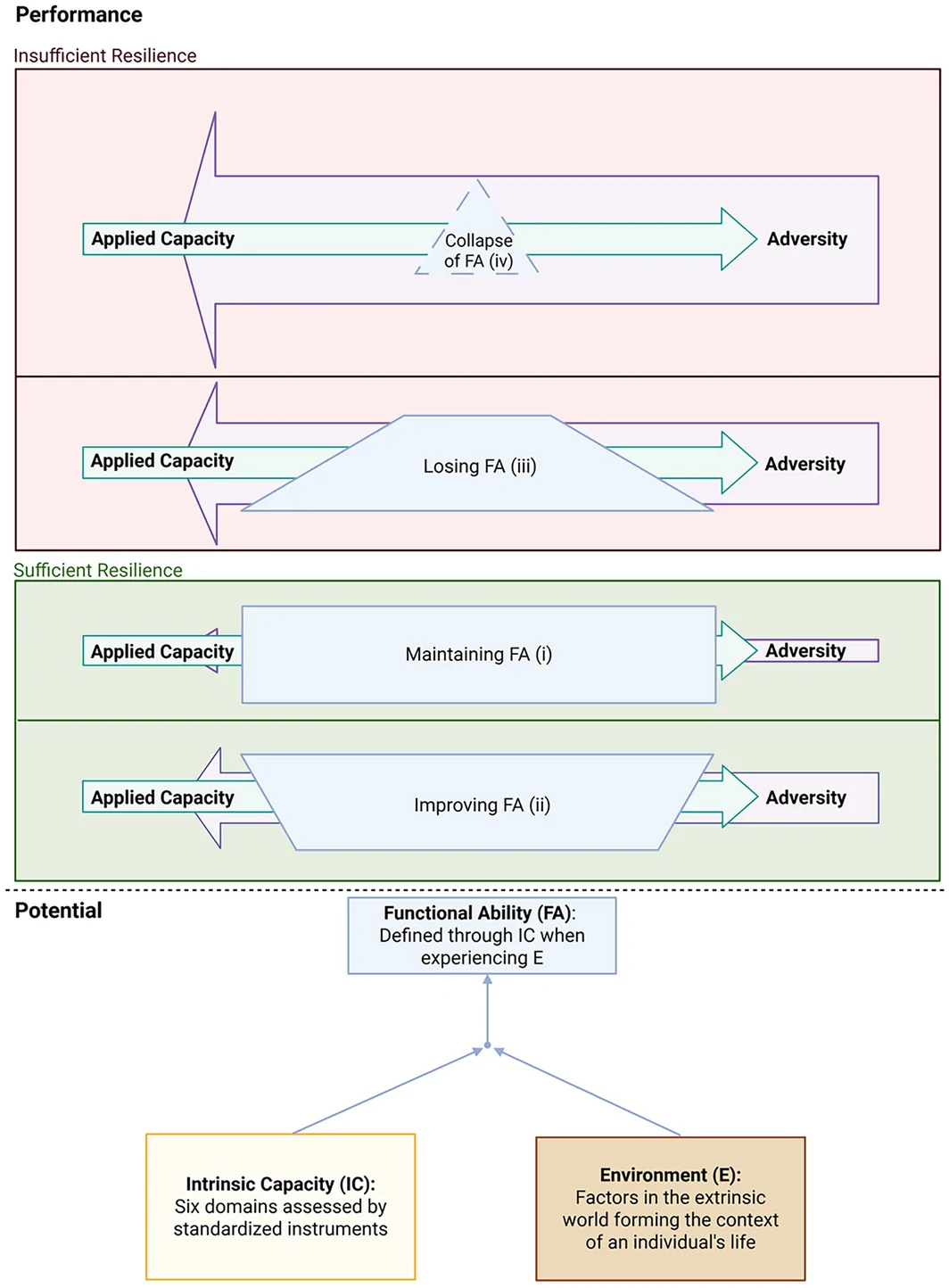
Overview of resilience in the context of functional ability under adversity. Above the dashed line (domain of performance), resilience becomes observable when adversity is encountered and determines whether FA is maintained or improved through the interaction of adversity and applied capacity—the resources an individual can actually draw on to meet a given adversity. Responses are shown for different levels of adversity (arrow thickness) relative to applied capacity: Where adversity is within applied capacity, FA is maintained (i); in the case where adversity becomes higher than applied capacity, an adaptation in the form of FA expansion is likely (ii); where adversity outweighs capacity, FA is lost (iii) or, in the extreme, collapses (iv). Resilience is thus sufficient in (i) and (ii), and insufficient in (iii) and (iv). Below the dashed line (domain of potential), intrinsic capacity (IC)—the composite of a person's physical and mental capacities, assessed by standardized instruments—together with the environment (E), the extrinsic factors forming the context of an individual's life, defines FA: The level of functioning achievable in the absence of adversity. Figure created with biorender.com.

With the onset of adversity, which can be represented by intrinsic or extrinsic stressors (Figure 2), resilience becomes assessable and determines how FA is maintained or improved. Through resistance, recovery, and adaptation, the system may hold function at its prior level or, in some cases, transfer it to a higher one (positive adaptation) (Udeh‐Momoh et al. 2025). Where adversity exceeds the limits of capacity, these mechanisms no longer suffice, and resilience becomes insufficient: function settles at a lower level (negative adaptation) or ultimately collapses. It is therefore the extent of resilience that determines how FA is expressed as performance (Figure 1).

**FIGURE 2:**
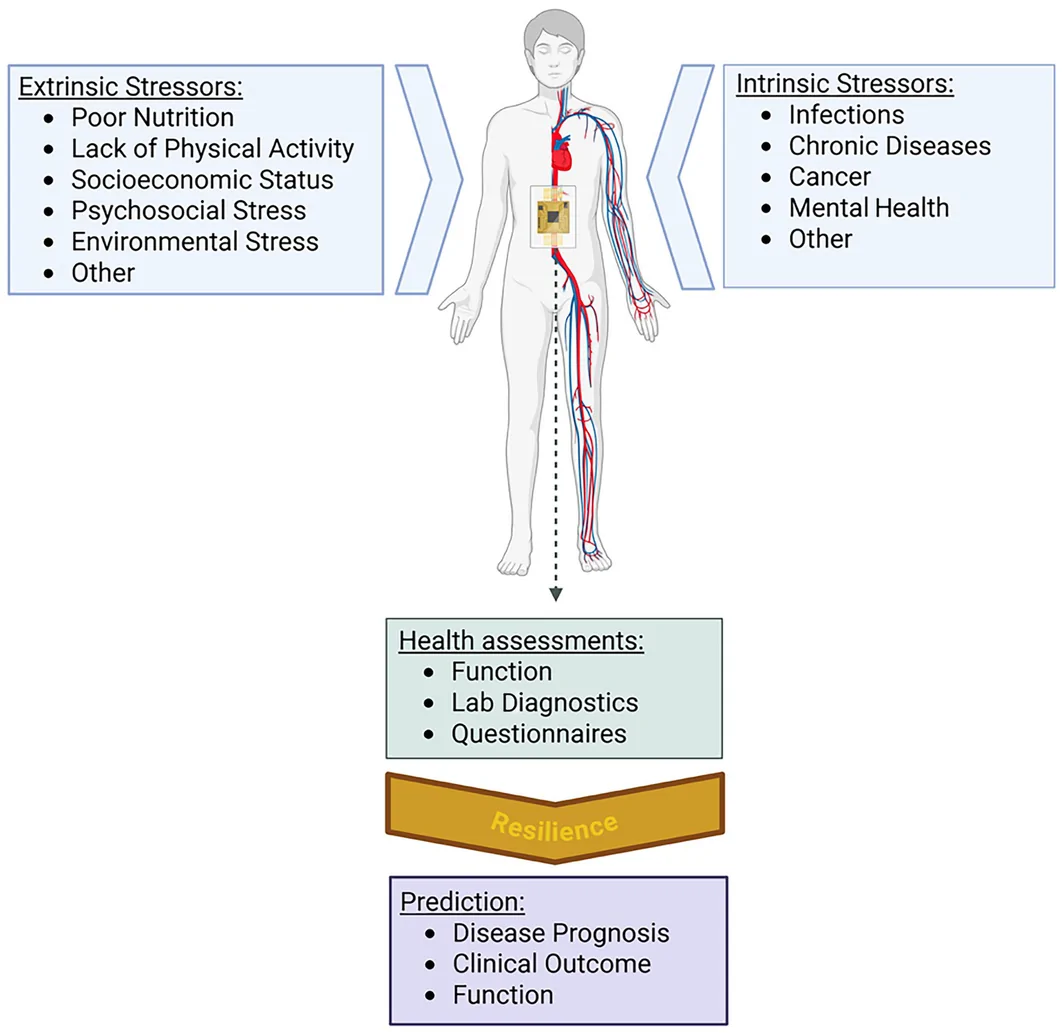
Stressors and resilience: The relation between adversity and resilience assessment for clinical application and outcome prediction. Figure created with biorender.com.

Resilience is not static: as individuals age, it declines, and the body increasingly struggles to maintain its integrity after perturbations (Promislow et al. 2022; Hadley et al. 2017). In complex systems, perturbations that are not followed by a perfect return to the equilibrium state often do not immediately lead to system collapse (e.g., organ failure or death) but rather to an adjusted stable state and a decrease in resilience (Wensink and Cohen 2021). Notably, such a decrease may remain undetectable in the absence of adversities—meaning that two individuals with differing levels of resilience might appear phenotypically indistinguishable until perturbed (Cohen et al. 2022; Varadhan et al. 2008). Resilience is thus a dynamic capability rather than a mere response strategy to crises, partially due to the flexibility (Udeh‐Momoh et al. 2025) and context‐dependent nature of physiological responses and can be assessed only indirectly, through the responses of biological systems and humans to adversity—the intrinsic and extrinsic stressors impacting the body (Colon‐Emeric et al. 2023; Johnston et al. 2015) (Figure 2).

Intrinsic stressors (Figure 2) are defined through internal processes in the organism at the physiological level, including chronic diseases and infections (Sierra 2020; Dou et al. 2020). These may act at two levels: chronically, as determinants of baseline intrinsic capacity (potential domain), and acutely, as perturbations that challenge resilience (performance domain). The risk of developing chronic diseases increases with age due to a decline in resilience (Whitson et al. 2016; Pyrkov et al. 2021), possibly mediated, for example, through pro‐inflammatory cytokines, persistent inflammation, and immune senescence (Ferrucci et al. 2020; Merchant et al. 2022). Conversely, the presence of adversity, such as disease, also affects the overall resilience level (Walston et al. 2023), indicating a bidirectional relationship between applied capacity, adversity, and ultimately impacting resilience (Figure 1). Beyond the (patho‐)physiological impact, mental aspects such as psychological adaptation and emotion regulation further support an individual's ability to respond to adversity, demonstrating that resilience is an active process rather than merely the absence of adversity, and can be strengthened by enhancing protective factors (Feder et al. 2009). Extrinsic stressors originate from factors outside of the individual and have an impact on it, including lifestyle, social, and environmental conditions. Extrinsic stressors' impact is directly influenced by how stressors are perceived and processed by the brain (Oken et al. 2015), a process also dependent on resilience level (García‐León et al. 2019). At the molecular level, the state of the transcriptome indicated by the transcriptional resilience was shown to be able to predict thermotolerance of *Caenorhabditis elegans* (Jovic et al. 2019), illustrating that stressor‐linked responses are measurable across biological scales (Box 1).

BOX 1 **Resilience across settings and the life course.**

**Table:** 

Mechanism	Setting	What is observed
Resistance	Outdoor workers (Habibi et al. 2024); Older adults (Tony Wolf et al. 2023) and vulnerable patient groups during heat waves (Hatvani‐Kovacs et al. 2016; Chen et al. 2025)	Magnitude of deviation in core temperature; time to return to homeostatic body temperature (Schorr et al. 2018)
Recovery	• Patients undergoing major abdominal surgery (Skořepa et al. 2024); Hospitalized patients assessed by serial handgrip (Tribolet et al. 2025)	Rate and completeness of functional return following the stressor
Adaptation	Prior thermal exposure (oysters) (Pereira et al. 2020)	Improved response or survival upon re‐exposure

Resilience and/or resources, depending on the definition, have been associated with clinical recovery (Merchant et al. 2022), lung pretransplant death/delisting (Bui et al. 2020), and postoperative delirium in older patients (Terrelonge et al. 2022).

Resilience can also be built within physiological boundaries. Regular physical activity reduces the risk of numerous chronic conditions, has cardioprotective effects, and improves mental health as well as well‐being (Chodzko‐Zajko et al. 2009), collectively supporting the maintenance of function under physiological stress. In children, resilience can be optimized through nutritional supplementation (Abdul Kari 2025), psychosocial stimulation (Yousafzai et al. 2013), and regulation of daily life stressors (DiCorcia and Tronick 2011); in workers, higher resilience accompanies better handling of demanding physical and psychological work environments (Shatté et al. 2017) and lower burnout rates (West et al. 2020; Castillo‐González et al. 2024). Over longer timescales, removing chronic exposure can restore lost capacity: quitting smoking before age 30 prevents over 97% of the excess mortality among UK women (Pirie et al. 2013).

What remains lacking is an operational approach to measuring resilience (Gijzel et al. 2019). Although the ICOPE programme operationalizes intrinsic capacity across its six domains (Table 1) through a structured screening and assessment pathway (WHO 2025), no equivalent framework captures the stressor‐linked recovery dynamics implied by the WHO definition of resilience. Several methods to quantify resilience have been proposed, including performance‐based instruments such as the Short Physical Performance Battery (Pedone et al. 2021), assessment of biological markers (e.g., cortisol levels (Aizpurua‐Perez et al. 2023)), stimulus–response patterns (Gijzel et al. 2019, 2017), and functional measures such as grip strength (Pepe et al. 2025). Herein, functional assessments are particularly relevant due to the strong interconnection between resilience and the level of physical capacity (San Román‐Mata et al. 2020; De Pauw et al. 2024). Performance‐based measures have accordingly been proven effective in predicting long‐term functional decline among older women with breast cancer (Owusu et al. 2017), and a specific condition of early fatigue in cancer patients undergoing radiotherapy (Strauss et al. 2007). In disease‐specific contexts, such assessments also enable the identification of treatment‐related risks and the evaluation of chemotherapy‐related toxicities in older cancer patients (Mohile et al. 2015). These findings underscore the need for standardized and validated tools to quantify resilience through objective measures. In this context, continuous, real‐time data, such as cardiorespiratory and physiological parameters, form the foundation.

## 2 Clinical Resilience

Capturing resilience requires following a person before, during, and after a challenge—something that has so far not been feasible in routine care. In its absence, clinical practice has relied on single‐time‐point assessments and clinical scores to estimate outcomes and disease or treatment trajectories. However, these established tools frequently provide static, condition‐specific snapshots (Cella et al. 2007), whereas person‐centered tools to identify individuals at health risk remain lacking (Carrasco‐Zanini et al. 2024). Although risk prediction tools exist for conditions like heart failure and stroke (e.g., the American College of Cardiology/American Heart Association 10‐year risk equation) (Carrasco‐Zanini et al. 2024), many health conditions—particularly in older and complex adults—lack comprehensive personalized models.

This limitation is compounded in advanced age, where people are commonly multimorbid, that is have several coexisting conditions or diseases that mutually influence each other, which affects patient needs and the clinical care organization (Gijzel et al. 2019; Blatch Armon et al. 2025; Wildiers et al. 2026). Beyond the requirements of each individual disease, the high potential for complexity of multiple co‐occurring diseases and their impact on everyday life needs to be addressed. Interactions of several co‐existing diseases (definition of the term “Multimorbidity” according to MESH, National Library of Medicine (US) 2018) can lead to conflicting disease management and almost insurmountable dilemmas for treating physicians (Battegay et al. 2017). Even cases with low disease complexity may be convoluted by patient factors such as functional status and psychosocial context. Although risk factors are likely to increase in number and impact during aging, not every person at risk is destined to become ill. In the context of multimorbidity (e.g., concurrent heart and kidney failure) (Skou et al. 2022), it is often unclear what initiated a health deterioration (Olde Rikkert et al. 2022). So far, healthcare systems, organizations, and research are ill‐equipped for such complex patients (Gijzel et al. 2019): care is often fragmented across specialists, leaving patients or families to coordinate and make decisions on their own, which may lead to worse outcomes, and responsibility is often unclear when various medical disciplines are involved. Co‐existing depression in multimorbid patients, for instance, is associated with longer hospital stays and higher readmission rates (Beeler et al. 2020), illustrating how unaddressed mental health compounds clinical complexity. Given that most biomarkers and clinical guidelines focus on single diseases or organ systems (Blatch Armon et al. 2025), a robust measurement capable of quantifying what sustains a person's resources, function, and survival could help shift fragmented organ‐specific care into a more comprehensive and patient‐centered approach aligned with value‐based health.

Patients undergoing major surgeries or systemic oncological treatments often face intraoperative health challenges, as well as prolonged recovery trajectories, where the ability to cope with physiological stress and regain function varies widely. Early identification and monitoring of individuals with reduced resilience could optimize clinical decision‐making and prompt person‐centered pre‐ and rehabilitation strategies, for example by building resilience before a clinical intervention, ultimately improving outcomes and reducing complications. Tribolet et al. (2025) showed that repeated handgrip measurements in malnourished hospitalized patients strongly predict mortality, supporting the idea that serial functional assessments reflect recovery capacity and resilience as well as potentially guiding pre‐ and rehabilitation strategies. Skořepa et al. (2024) showed that prehabilitation was associated with a 44% lower risk of developing severe complications following major abdominal surgery. Notably, serial functional assessment and prehabilitation are already established in geriatric and perioperative practice; we invoke them here not as novel applications but as evidence that stressor‐linked, dynamic measures carry prognostic information that static baseline scores might not.

Clinical resilience is therefore not another static screening instrument, nor a replacement for frailty assessment, physical‐performance testing, intrinsic capacity assessment, multimorbidity‐based risk stratification, or comprehensive geriatric assessment. These established assessments predominantly characterize baseline vulnerability, functional capacity, and potentially modifiable deficits. Clinical resilience instead links a defined stressor to the magnitude of the perturbation, the resistance to decline, the rate and completeness of recovery, and the recovery relative to that expected from baseline characteristics and stressor severity. It could thus distinguish patients with similar baseline frailty or physical‐performance scores but divergent recovery trajectories, support patient‐centered prediction of whether, how quickly, and to what level an individual is likely to recover, and permit prognosis to be updated during the clinical course. However, evidence that resilience measures add meaningful predictive or decision value beyond established assessments remains preliminary; direct comparative validation and clinical‐impact studies are required before routine implementation.

For clinical implementation, we propose that clinical resilience might be conceptualized along two complementary lines. The first line concerns the body's capacity to resist perturbations and recover from significant stressors, potentially leading to a diseased state. This is related to one disease or to becoming multimorbid by developing an additional one. The second pertains to an individual's ability to recover and adapt when already affected by illness and undergoing medical interventions (stressor/adversity), such as major surgery or systemic drug therapy (Figure 3). The two lines are distinguished not by biology but by the clinical character of the stressor, and they map onto established domains: the first onto the concern of geroscience and preventive medicine with disease incidence and the maintenance of health; the second onto geriatric and perioperative medicine. They differ, however, in tractability. In the first line, the stressor is typically unscheduled and often recognized only in retrospect, so that no pre‐stressor baseline has been acquired; in the second, it is scheduled, its type and severity can be graded, and a baseline can be established prospectively. The evidence discussed above falls almost entirely within the second line, as does the stimulus–response paradigm proposed by Varadhan et al. (2008) We therefore regard the second line as the setting in which clinical resilience can first be specified and tested, and the first as the more challenging extension.

**FIGURE 3:**
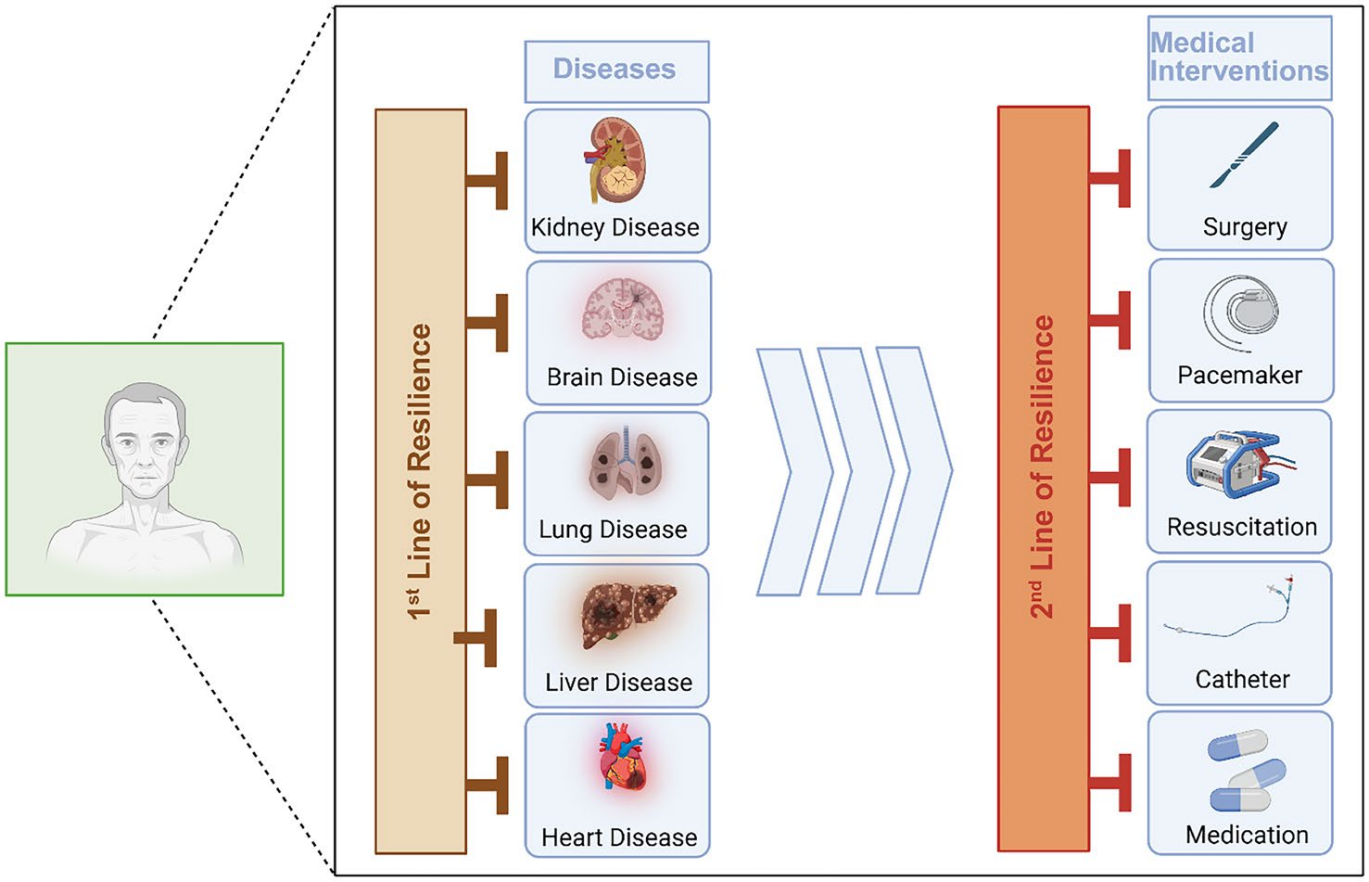
The two lines of resilience in clinical medicine: The application of resilience in clinical medicine is built on two lines. The first line covers the process of becoming diseased, in general, thus providing a tool to identify and manage general disease risk. The second line concerns the ability of a person to overcome an indicated but potentially harmful medical intervention—such as major surgery and systemic pharmacotherapy—ultimately supporting the understanding and optimization of clinical outcomes. The diseases and interventions shown are illustrative examples and not a comprehensive list. Figure created with biorender.com.

Clinical resilience can be operationalized longitudinally relative to an individual's pre‐stressor baseline and the characteristics of the stressor. As a proposed framework for its measurement, resistance could be represented by the magnitude of deviation from baseline during or immediately following the stressor; recovery by the rate or time course of return toward baseline; and adaptation by a change in the response to subsequent comparable stressors. These dimensions could be quantified using appropriate functional, physiological, or molecular measures without prespecifying a single biomarker or threshold. This provides a testable framework while allowing the specific signals and clinically meaningful thresholds to be established through future validation.

## 3 Wearable Technologies: A Scalable Window Into Clinical Resilience

A meaningful clinical resilience assessment, so defined, requires repeated measurements rather than a single time point to determine a function over time. It begins with a pre‐adversity baseline, characterizing the resources an individual can draw on, which is what intrinsic capacity denotes. It then follows the period of exposure to adversity, of sufficient duration to capture the response (Brasier, Wang, Dincer, et al. 2024), during which resilience is expressed as the maintenance or improvement of functional ability. Within these periods, resistance, recovery, and adaptation can then be assessed relative to the trajectory expected from baseline characteristics and stressor severity, rather than in absolute terms, and tested against clinical outcomes specified in advance, such as mortality, length of hospitalization, patient‐reported outcomes (Conroy and van Oppen 2023), and costs.

From fitness tracking to advanced biosensing, wearable technologies now monitor a wide range of health‐related parameters across the life course: biophysical (e.g., heart rate and movement), biochemical (e.g., sweat composition), and acousto‐mechanical (e.g., pulse waveform), alongside features of the environment (e.g., temperature). Because many of these measurements are acquired continuously in the person's own environment, they can track parameters not only at rest but through exposure to adversity and the period that follows, yielding context‐derived health information on the maintenance and recovery of functional ability (Brasier, Mahato, et al. 2025) (Figure 4). Increasingly, these systems combine multiple sensing modalities and apply machine learning (ML) (Bian et al. 2026) to extract clinically relevant patterns, pointing toward a shift from passive logging to personalized, predictive assessment.

**FIGURE 4:**
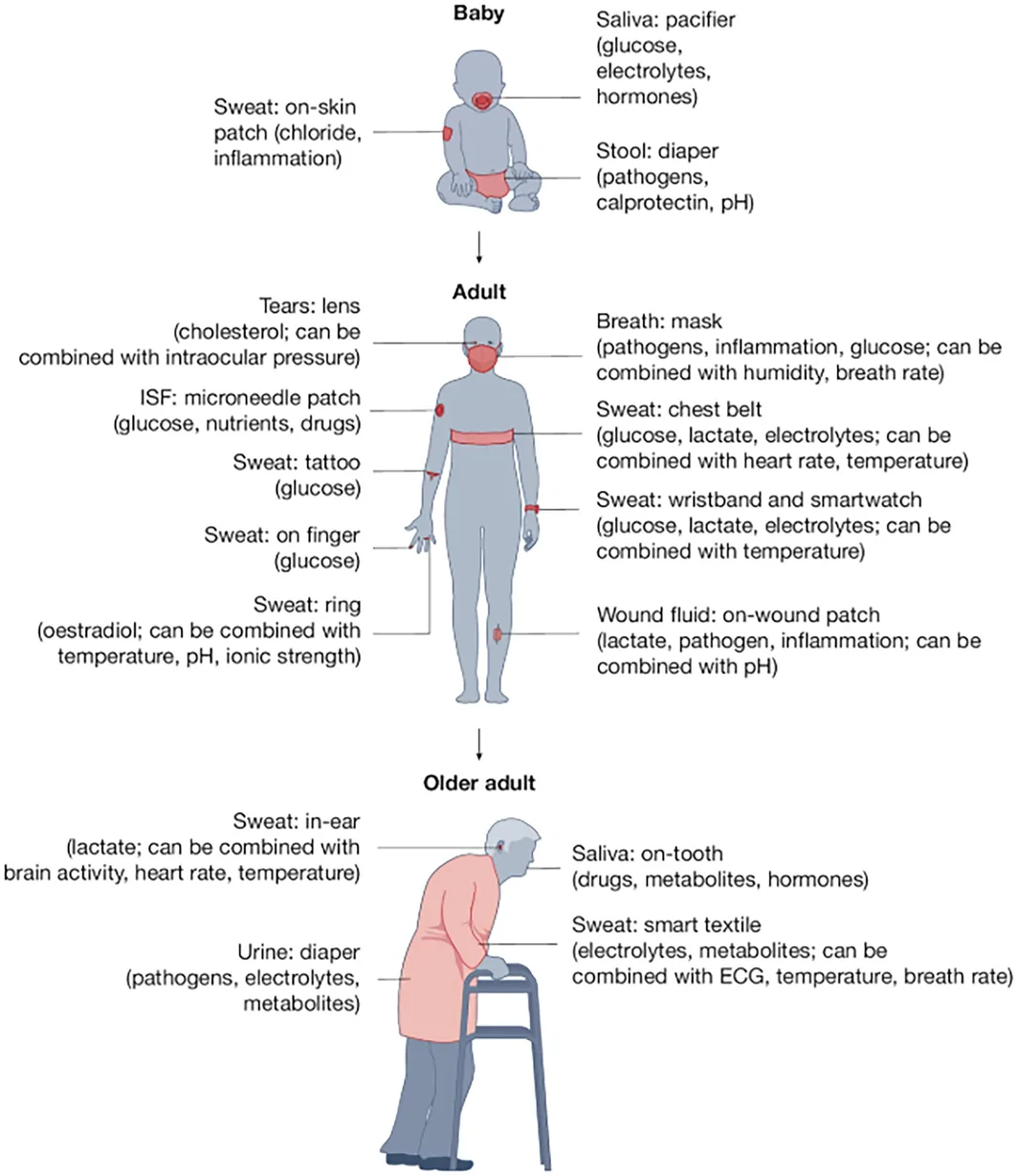
Assessing health and disease: Health monitoring through wearable devices across the life cycle, ranging from pacifiers analyzing saliva to sweat analysis integrated into hearing aids (Replicate from Brasier, Wang, Gao, et al. 2024).

Wearable measurements can be grouped by the function whose maintenance they track: cardiovascular function through heart rate, heart rate variability, and blood pressure; thermoregulation through core body temperature and sweat rate; and rest and recovery through sleep architecture and activity rhythms. These functions are fundamental to maintaining and improving FA under adversity. Several also map onto domains of IC (Figure 1; Table 1): activity and gait measures onto locomotor capacity, and cardiorespiratory and metabolic measures onto vitality, for which no single accepted measurement set exists, and candidate indicators spanning physiological reserve, energy and fatigability, and nutritional or metabolic state have been proposed (ARPA‐H Convened Expert Working Group 2026).

The translational readiness of technologies varies substantially. To our knowledge, no integrated wearable framework has yet been clinically validated for comprehensive resilience assessment; current modalities are therefore candidate data‐acquisition tools for developing and testing resilience measures, rather than validated resilience tests, and they differ in maturity (Table 2). Among established modalities—clinically available or routinely used for their original measurement purpose—wrist‐worn accelerometry can characterize sleep–wake cycles and activity rhythms associated with biological aging, morbidity, and mortality (Shim et al. 2024), while photoplethysmography (PPG) and continuous glucose monitoring (CGM) are likewise established for their original measurement purposes (Brasier, Mahato, et al. 2025; Brasier, Wang, Gao, et al. 2024). Emerging biosensors integrate microfluidics and electrochemical sensors to measure analytes such as glucose (Gao et al. 2016), lactate (Gao et al. 2016), cortisol (Torrente‐Rodríguez et al. 2020), and inflammatory proteins (Tu et al. 2023). Platforms like the 3D‐printed e^3^‐skin (Song et al. 2023) illustrate an exploratory multimodal system combining sweat analysis, electrophysiological signals, and machine‐learning‐based interpretation.

**TABLE 2:** Technology readiness and translational roadmap of candidate resilience‐assessment modalities.

Roadmap stage	Representative modalities	Technology readiness	Primary translational hurdles
Established	PPG, Accelerometry, Continuous Glucose Monitor (CGM), Handgrip dynamometry	Available or routinely used for constituent measurements; not validated for resilience assessment	Prospective validation against predefined stressors, recovery metrics, and clinical outcomes; Incremental value beyond geriatric assessments; Workflow integration and usability
Emerging	Targeted sweat sensors (e.g., Lactate, Cortisol, C‐Reactive Protein (CRP))	Clinical/analytical validation in progress; not validated for resilience assessment	Standardization sampling and calibration; Longitudinal reliability; Analyte‐specific outcome validation; Inter‐ and intra‐individual variability
Exploratory	Untargeted MS‐to‐wearable pipeline, Multimodal e^3^‐skin	Discovery‐stage or hypothesis‐generating	End‐to‐end demonstration; Hardware miniaturization; Establishing long‐term clinical endpoints and regulatory pathway

*Note:* Technologies are grouped by roadmap stage: Established measurement modalities are clinically available or routinely used for their original measurements but not validated as integrated resilience assessments; Emerging modalities are under clinical or analytical validation; Exploratory approaches are discovery‐stage or hypothesis‐generating. Most modalities shown are wearable or point‐of‐care modalities; untargeted mass spectrometry (MS) is included as a complementary, non‐wearable platform that represents the molecular discovery pipeline feeding future wearable development. Colour shading (green, orange, red) follows a traffic‐light scheme to indicate decreasing levels of technology readiness.

While promising, many available wearable systems have a narrow target scope and measure only a few isolated—often physical—parameters (even when multiplexed) and therefore may miss unanticipated molecular changes, especially those linked to aging and resilience. Untargeted mass spectrometry (MS), particularly MS‐based metabolomics and small‐molecule profiling, can complement wearable sensing by revealing stress‐responsive molecular changes that may not be captured by current targeted devices, and is particularly suited to resilience for a specific reason: resilience is defined by the dynamic response to adversity, which can unfold over minutes to hours and small molecules and metabolites turn over on faster timescales, from seconds to minutes, enabling detection of the immediate, proximal molecular changes that drive it. By contrast, epigenetic clocks reflect cumulative long‐term biological adaptation rather than acute perturbation dynamics, and proteomic changes are generally constrained by slower protein turnover rates. MS‐based metabolomics therefore provides one of the most temporally proximal molecular readouts of stress‐recovery dynamics relevant to resilience assessment (Belsky et al. 2022; Argentieri et al. 2024; Patti et al. 2012; Huang et al. 2025).

Unlike targeted approaches, untargeted MS supports the hypothesis‐free identification of biomarkers potentially associated with physiological recovery. When paired with ML algorithms capable of extracting patterns from high‐dimensional datasets, this approach can help identify molecular signatures that characterize an individual's stress‐response phenotype. Rather than serving as a continuous monitoring tool itself, untargeted MS functions as an upstream “discovery engine,” where ML models distill complex MS spectra into a refined set of informative, analytically tractable targets. These ML‐derived panels then help guide downstream sensing requirements, such as required sensitivity and dynamic range for targeted wearable implementation (Kim et al. 2026).

Depending on the physicochemical properties of the identified analytes, these formats could include epidermal sweat patches, microneedle‐based interstitial fluid sensors, or other affinity‐ or electrochemistry‐based wearable platforms (Lipani et al. 2018; Ye et al. 2023; Tu et al. 2025; Tehrani et al. 2022). In this sense, the translational logic is analogous to that of CGM in its continuous monitoring paradigm, but distinct in that the specific analyte panels for resilience are empirically identified through untargeted MS discovery rather than being pre‐defined.

Together, these advances suggest a new framework: untargeted MS—as one of various possible tools—identifies molecular targets; ML integrates high‐dimensional molecular and physiological data to identify predictive patterns and generate testable hypotheses; and specifically functionalized wearables enable real‐time, personalized monitoring of these analytes (Chen, Ding, and Wang 2023). In aging, such an integrated strategy may help move static assessments toward a more dynamic and mechanistically informed evaluation of how individuals respond to and recover from adversity.

This full pipeline has not yet been shown end‐to‐end. Untargeted MS‐based discovery and targeted wearable sensing have been reported only as separate steps, so the integrated approach remains a proposal rather than an established capability. Its clinical value for developing resilience assessments in older adults in particular remains to be established, and at present, this should be regarded as a hypothesis‐generating direction rather than a validated approach. Identifying which analytes could serve this purpose is therefore a priority requirement for translation.

## 4 Next Steps, Challenges, and Opportunities

To establish and unlock the full potential of clinical resilience, we have identified 5 key challenges that must be addressed (Gijzel et al. 2019; Ruckstuhl et al. 2023). Foremost, comprehensive sets of candidate biomarkers that accurately reflect physiological processes associated with clinical resilience must be identified. For this, noninvasive and continuous sampling methods are particularly promising, allowing for the collection of health information with a high degree of time resolution. Next to broadly available physical biomarkers for vital sign monitoring, we propose that sweat is a particularly suitable and underutilized modality for adding biochemical biomarker identification. Sweat has emerged as a valuable biofluid, providing access to a wide range of molecular information (Brazaca et al. 2026). Many biomarkers detectable in sweat overlap with established clinical markers found in serum, highlighting sweat's potential for applications in clinical medicine (Niederberger et al. 2023; Brasier and Eckstein 2019; Brasier et al. 2023). However, it remains unclear whether sweat and serum convey equivalent health information. Beyond its molecular constituents, sweat also provides physical information—such as sweat rate and composition dynamics—which can serve as indicators of physiological regulation and stress responses (Tu et al. 2025). Together, these features position sweat as a multifaceted source of data that may reflect health status, disease, and ultimately resilience. Recent work further highlights the potential of using alternative body fluids, such as sweat, to extend monitoring beyond established environmental measures (Brasier, Niederberger, et al. 2025). Nevertheless, challenges remain due to the inherent inter‐ and intra‐individual variability of sweat composition and production. Among the various non‐/minimal invasive blood‐alternative body fluids accessible through wearable devices, such as saliva, breath, and interstitial fluid (ISF) (Brasier, Wang, Gao, et al. 2024), sweat stands out as a particularly promising medium as it reflects systemic physiological responses, including those of the cardiovascular (Epstein and Yanovich 2019) and neurological systems (Brasier, Sempionatto, Bourke, et al. 2024), and serves as a general indicator of health status (Noé et al. 2024).

Beyond this, it is essential to establish comprehensive links between biological measures and clinical outcomes to demonstrate their relevance and predictive value. This involves longitudinal studies that track individuals over time to understand health trajectories of health assessments, functional decline, recovery following adversity, and patient‐related outcome measurements (Gijzel et al. 2019). Establishing these associations is critical for validating measures as reliable indicators of resilience rather than incidental biological variation. Furthermore, integrating multi‐omics data with clinical endpoints can enhance our understanding of the underlying biological pathways, allowing for a more nuanced characterization of resilience across diverse populations and aging phenotypes.

As a one‐size‐fits‐all wearable device to assess clinical resilience is not yet available, device development and integration bear significant hurdles and should be conducted in an integrative approach (Brasier, Domenghino, et al. 2025). To date, various solutions and companies provide devices, mostly with proprietary application programming interfaces, software, and algorithms. Transforming digital infrastructures, allowing, for example, the seamless integration of multiple devices into secured, on‐premises servers (Brasier et al. 2020), is in development and holds promise to provide clinicians and patients with usable technological solutions. Further, connectivity and laboratory‐independence of wearable devices allow for fostering hospital‐at‐home (Pandit et al. 2024), clinical care at home, with high potential to reduce economic burden on healthcare systems around the world. However, the use of wearable devices to stream health data from a patient's home to the treating physician requires a basic level of digital literacy, which may be limited in patients of advanced age. Thus, tailored education to the respective needs is essential (Xu et al. 2024). Further, enabling such digital connectivity effectively introduces an external point of access to the private home (Brasier, Mahato, et al. 2025).

From a clinical developmental perspective, a subsequent challenge lies in translating these findings from controlled research environments into clinically applied tools. This process requires comprehensive clinical testing to ensure reliability, reproducibility, and relevance across diverse patient populations and real‐world settings. Integration into existing clinical workflows must be seamless, minimizing disruption while enhancing decision‐making processes. Additionally, regulatory approval pathways must be navigated from the beginning, requiring alignment with current policy frameworks and compliance with evolving standards for data security, patient privacy, and ethical oversight. Effective translation also hinges on interdisciplinary collaboration, bringing together clinicians, researchers, technologists, and policymakers to foster adoption, ensure equity in access, and ultimately improve health outcomes through precision approaches to aging and resilience.

A further challenge is integration into evolving models of care delivery. Healthcare for older adults is costly, driven by increased longevity, chronic conditions, and high end‐of‐life care (Jones and Dolsten 2024) costs, with women facing higher costs owing to longer life expectancy and greater use of long‐term care (Chen, Zhao, et al. 2023; Carvalho et al. 2019). Over the past two decades, cost‐effectiveness strategies have accordingly shifted their focus from treatment toward prevention and health education to reduce the occurrence of chronic diseases (Johnston 2020). New care delivery models such as home‐based care, telehealth (Steindal et al. 2023), and digital therapeutics offer increased efficiency and reduced reliance on expensive hospital care (Carrera et al. 2024; Roberts et al. 2023). Such strategies, focused on building health through prevention, integration, and value‐based care, may support long‐term sustainability and improve the quality of life for our aging populations. Integrated into these emerging care models and subject to clinical validation, the assessment of clinical resilience could contribute to how health and care are understood for people of advanced age.

## 5 Conclusion

Clinical resilience may offer a complementary metric in the assessment of biological aging, potentially shifting the paradigm from static risk models to dynamic assessments of adaptive capacity and building health. Understanding how well individuals cope with physiologically stressful conditions could contribute to a more context‐driven view of health and disease across the lifespan.

Resilience represents a complex, integrative functional construct spanning physiological, clinical, socioenvironmental, and mental health‐based dimensions. The growing availability of wearable and connectable devices may provide tools to support the development of multimodal resilience assessments, although their validity and clinical utility for this purpose remain to be established. If validated, such an approach could help inform clinical care for aging populations by supporting outcome prognosis, targeted interventions, and person‐centered healthcare, ultimately generating value at both individual and societal levels. Achieving this vision will require multidisciplinary collaborations to further conceptualize and demonstrate resilience's clinical potential and added value.

## Author Contributions

Conceptualization: V.M.G., A.M., C.Y.E., N.B.; Funding acquisition: N.B., C.Y.E., S.A., R.R., W.G., J.G.; Supervision: N.B.; Visualization: V.M.G., N.B.; Writing – original draft: V.M.G., E.M., M.‐J.K., A.M., S.J., G.F.S., E.R., C.R.J., E.B., R.M.R., S.A.; Writing – review and editing: F.M., C.Y.E., W.G., J.G., N.B.

## Funding

This work was supported by Schweizerischer Nationalfonds zur Förderung der Wissenschaftlichen Forschung, 10000659. National Science Foundation, 2444815.

## Conflicts of Interest

With no relation to the current manuscript, C.Y.E. is a shareholder and cofounder of AVEA Life AG and Lichi3 GmbH. C.Y.E. is a shareholder and employee of Novartis. A.M. is an employee of Novartis while conducting a shared PhD degree between Novartis and ETH Zurich. V.M.G., E.M., M.‐J.K., S.J., F.M., G.F.S., E.R., C.R.J., E.B., R.M.R, S.A., J.G., W.G., N.B. declare no conflicts of interest.

## Data Availability

This work does not report original research; therefore, no new data were generated or analysed, and no data are available for sharing.
